# Spinal Cord Motion in Degenerative Cervical Myelopathy: The Level of the Stenotic Segment and Gender Cause Altered Pathodynamics

**DOI:** 10.3390/jcm10173788

**Published:** 2021-08-25

**Authors:** Katharina Wolf, Marco Reisert, Saúl Felipe Beltrán, Jan-Helge Klingler, Ulrich Hubbe, Axel J. Krafft, Nico Kremers, Karl Egger, Marc Hohenhaus

**Affiliations:** 1 Medical Center, Department of Neurology and Neurophysiology, Faculty of Medicine, University of Freiburg, 79106 Freiburg im Breisgau, Germany; saul.beltran@uniklinik-freiburg.de; 2 Medical Center, Department of Radiology, Medical Physics, Faculty of Medicine, University of Freiburg, 79106 Freiburg im Breisgau, Germany; marco.reisert@uniklinik-freiburg.de (M.R.); axeljoachim.krafft@siemens-healthineers.com (A.J.K.); 3 Medical Center, Department of Neurosurgery, Faculty of Medicine, University of Freiburg, 79106 Freiburg im Breisgau, Germany; jan-helge.klingler@uniklinik-freiburg.de (J.-H.K.); ulrich.hubbe@uniklinik-freiburg.de (U.H.); marc.hohenhaus@uniklinik-freiburg.de (M.H.); 4 Medical Center, Department of Neuroradiology, Faculty of Medicine, University of Freiburg, 79106 Freiburg im Breisgau, Germany; nico.kremers@uniklinik-freiburg.de (N.K.); karl.egger@uniklinik-freiburg.de (K.E.); 5 Department of Radiology, Tauernklinikum Zell am See/Mittersill, 5700 Salzburg, Austria

**Keywords:** degenerative cervical myelopathy, phase-contrast MRI, automated segmentation, gender, convolutional neural network

## Abstract

In degenerative cervical myelopathy (DCM), focally increased spinal cord motion has been observed for C5/C6, but whether stenoses at other cervical segments lead to similar pathodynamics and how severity of stenosis, age, and gender affect them is still unclear. We report a prospective matched-pair controlled trial on 65 DCM patients. A high-resolution 3D T2 sampling perfection with application-optimized contrasts using different flip angle evolution (SPACE) and a phase-contrast magnetic resonance imaging (MRI) sequence were performed and automatically segmented. Anatomical and spinal cord motion data were assessed per segment from C2/C3 to C7/T1. Spinal cord motion was focally increased at a level of stenosis among patients with stenosis at C4/C5 (*n* = 14), C5/C6 (*n* = 33), and C6/C7 (*n* = 10) (*p* < 0.033). Patients with stenosis at C2/C3 (*n* = 2) and C3/C4 (*n* = 6) presented a similar pattern, not reaching significance. Gender was a significant predictor of higher spinal cord dynamics among men with stenosis at C5/C6 (*p* = 0.048) and C6/C7 (*p* = 0.033). Age and severity of stenosis did not relate to spinal cord motion. Thus, the data demonstrates focally increased spinal cord motion depending on the specific level of stenosis. Gender-related effects lead to dynamic alterations among men with stenosis at C5/C6 and C6/C7. The missing relation of motion to severity of stenosis underlines a possible additive diagnostic value of spinal cord motion analysis in DCM.

## 1. Introduction

The anatomical degenerations of the cervical spine, which may lead to the syndrome of degenerative cervical myelopathy (DCM) are well established (e.g., disc protrusions, ossification of ligaments, etc.) [1,2,3,4]. While relevant spinal canal degeneration may occur without any objective clinical signs or symptoms [1,5,6,7], further parameters may help to identify those at risk in developing cervical myelopathy.

Recent findings based on phase-contrast MRI (PC-MRI) have demonstrated significantly increased craniocaudal spinal cord motion among patients with degenerative cervical myelopathy (DCM) at the most commonly affected segment C5/C6 [8,9,10,11,12]. The increase of motion was demonstrated to be a focal phenomenon specifically at the stenotic segment C5/C6 [10,12], and also at the stenotic segment C4/C5 in a small group of four patients [13]. The spinal cord motion at more cranial segments remained unaffected [10,12].

Clinical impairment correlated to increased spinal cord motion within a small cohort [10]. Dynamic strain on spinal cord tissue was demonstrated and supports the conclusion of possible pathodynamic relevance [12]. To date and in contrast to the expected dynamic behavior, the extent of spinal cord motion cannot yet be associated to measurements of the severity of spinal stenosis at C5/C6 reflected by the compression ratio (*n* = 12) [10], or the adapted maximum canal compromise (aMCC; *n* = 29) [12]. This missing relationship indicates the need of either further refinements of anatomical assessments or the existence of influencing factors beyond local anatomy. Thus, MRI-based measurements of spinal cord dynamics may provide additive diagnostic information.

As there are many uncertainties regarding the spontaneous course, and consecutively, the treatment decision of mildly affected or multimorbid DCM patients, these new quantitative, non-invasively, and reliably assessable PC-MRI parameters [10,11,12,13] may be of future interest in the clinical decision-making process. Still, at the current state of basic research, there are many unanswered questions concerning the dynamic behavior of the cervical spinal cord and its influencing factors.

Based on known segmental differences of spinal cord motion across the cervical spine in healthy controls [11], it remains unclear whether spinal cord motion pattern in DCM differ from one stenotic cervical segment to another and how segmental spinal cord motion is affected by age, gender, and extent of stenosis.

We hypothesized that we could reproduce similar patterns of spinal cord motion at the different levels of cervical stenosis among DCM patients presenting with monosegmental stenosis. We assumed non-significant effects of age and gender on spinal cord motion. Also, we hypothesized to find interactions of spinal cord motion to automated assessments of spinal canal compression.

## 2. Methods

### 2.1. Study Design

We report a monocentric, prospective, matched-pair-controlled study. The first consecutive eighty patients from our ongoing longitudinal trial on DCM were analyzed (German registry of clinical trials, number: DRKS00012962). Patients were grouped according to the level of relevant cervical stenosis (C2/C3, C3/C4, C4/C5, C5/C6, and C6/C7). Relevant stenosis was defined as depleted cerebrospinal fluid (CSF)-space anterior and posterior or marked compression of the spinal cord visually diagnosed in T2-weighted MRI; mild to moderate degeneration at other segments not fulfilling these criteria were accepted.

Per group, each patient was matched one to one by age and gender to a healthy control, which we extracted from our database (German registry of clinical trials, number: DRKS00017351). Recruitment procedures as well as in- and exclusion criteria have been reported previously [12,14]. In short, patients were required to present at least mild symptoms (e.g., clumsy hands, bilateral non-radicular paresthesia, and hyperreflexia) due to monosegmental relevant cervical spinal stenosis. Clinical severity was scored via the modified Japanese Orthopedic Association (mJOA) score [15], and number of patients entering decompressive surgery was recorded. A maximum mJOA score of 18 points was accepted, as certain signs of spinal cord affection do not necessarily lead to a reduced score (e.g., hyperreflexia or intermittent hypesthesia). Controls were required to have no history or signs or symptoms of DCM and no incidental relevant cervical stenosis within the following MRI.

Patients with conflicting neurological symptoms, due to e.g., carpal tunnel syndrome, and controls volunteering with unaware neurological symptoms, were prospectively excluded by an interview, a neurological exam, and, if needed, by electrophysiological measurements before admission to the study.

Data was collected between June 2018 and February 2021. Data acquisition and analysis was performed in compliance with protocols approved by the Ethical Committee of the Albert-Ludwigs-University Freiburg (ethical approval numbers: 261/17, 338/17). Written informed consent was obtained from all participants prior to study.

### 2.2. Imaging Protocol

Each participant received one MRI scan (3T, SIEMENS MAGNETOM Prisma, SIEMENS Erlangen, Germany). This included a 3D T2 SPACE sequence for analysis of anatomical parameters (spatial resolution 0.64 × 0.64 mm^2^ × 1.0 mm, TR 1500 ms, TE 134 ms, Flip angle 105°, GRAPPA factor: 3, acquisition time 3:53 min) and a prospectively ECG-triggered PC-MRI sequence for detection of craniocaudal motion in sagittal orientation covering vertebra C1 to T1 (spatial resolution 0.62 × 0.62 mm^2^ × 3 mm, FoV 200 × 200 mm^2^, TR = 31.8 ms, TE = 7.75 ms, flip angle 15°, bandwidth 488 Hz/Pixel, velocity encoding parameter 5 cm/s, PEAK-GRAPPA, acquisition time: approximately 2 min depending on the heart rate.). An average of 40 timepoints per heartbeat and individual was gained. During the execution of the PC-MRI, the average duration of the heartbeat (HB) per individual was automatically recorded. Thus, individual data curves of velocity (cm/s) over time (s) can be resolved and used for further derivatives.

### 2.3. MRI Data Processing

**Figure 1:**
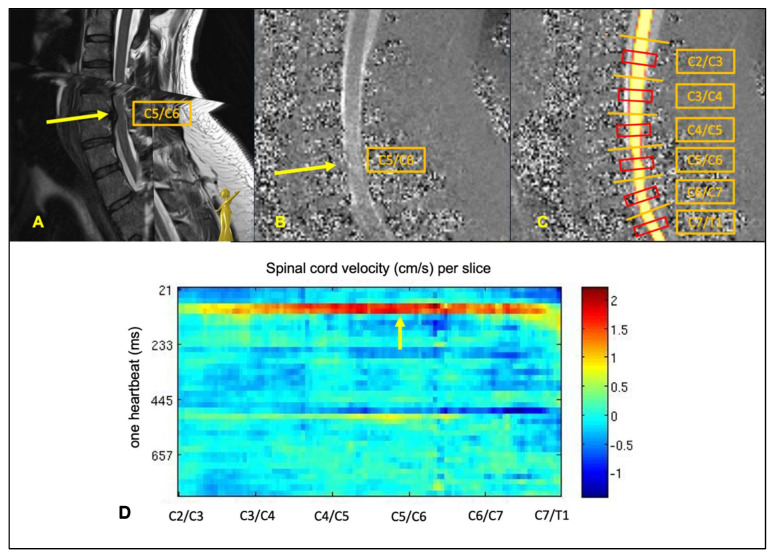
Example of spinal cord motion assessments within the current data processing pipeline. Top row (**A**): 3D T2w SPACE sagittal image of a patient with stenosis at C5/C6 (yellow arrow). (**B**): one exemplary phase-contrast image of the same patient within one heartbeat; the yellow arrow points onto the light grey colored spinal cord that reflects the focally increased craniocaudal spinal cord motion compared to the darker grey colored spinal cord motion at the surrounding segments. (**C**): segmentation of the phase-contrast image of the same patient, red squares demonstrate the ROIs per cervical segment covering 1/3 of the intervertebral space. (**D**): example of the spinal cord velocity plot of the same patient demonstrating color-coded spinal cord velocities (cm/s) (right side) per slice (*x*-axis) and per assessed time point (*y*-axis) during one heartbeat (ms).

Automated segmentation was performed by trained hierarchical, deep convolutional neural networks (CNN) implemented within an automated data processing pipeline using the medical imaging platform Nora [16]. The details on the trainings of the CNNs, and the data processing pipeline including segmentation, phase-drift correction method, and setting of the regions of interest (ROI) has been described previously [12]. The implementation of the CNNs was similar as reported by Zhao et al. [17]. In short, different CNNs were trained for segmentation of anatomic data (CSF-space and spinal cord cross sectional area (CSA)) based on the 3D T2-weighted sequence, and for segmentation of the spinal cord for analysis of dynamic data based on the phase-contrast sequence (example Figure 1). ROIs were generated covering the central 1/3 of the spinal cord / CSF-space between two cervical vertebra bodies (Figure 1). In total, six ROIs were analyzed per individual: C2/C3, C3/C4, C4/C5, C5/C6, C6/C7, and C7/T1 (Figure 1).

All phase–contrast images were inspected visually upon artifacts (e.g., movement, metal, infoldings, and flow-artifacts by vessels) before entering further analysis. Nine patients were excluded because of overall MRI artifact due to movement or infoldings. Six patients were excluded due to drop out during the MRI scan (*n* = 1), withdrawal of consent (*n* = 1), and detection of multisegmental relevant stenosis in the study scan (*n* = 4). In three cases, dynamic parameters at C2/C3 were excluded due to a flow-artifact (one per group with stenosis at C3/C4, C5/C6, C6/C7, respectively); dynamic parameters at segment C2/C3 and C3/C4 were excluded due to an artifact within one case with stenosis at C4/C5.

### 2.4. PC-MRI Parameters

**Figure 2:**
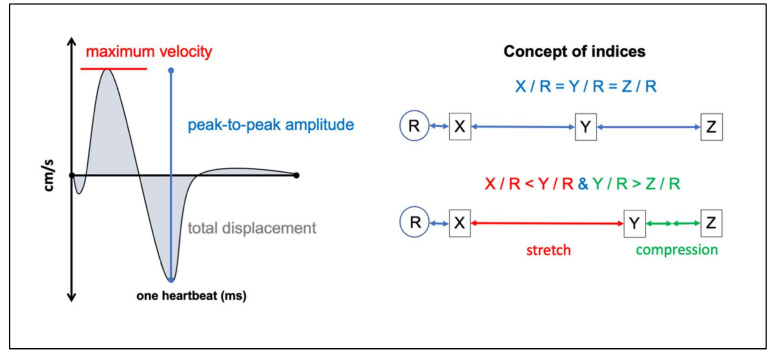
Schematic illustration of the dynamic spinal cord motion parameter based on the approximated 40 velocity values per individual plotted over one heartbeat (left side). Maximum velocity (red) refers to the highest positive (craniocaudal) velocity within the curve. Peak-to-peak-amplitude (blue) addresses the maximum positive (caudal) and negative (cranial) velocity within the curve and therefor adds further information on the extent of the motion. The total displacement (grey) comprises information of the entire curve (addition of the area under the curve irrespective of algebraic signs). Indices allow information beyond general group effects by intra-individual referencing. It minimizes possible general biodynamic confounders and gives information on possible strains (right side). If a point Y in relation to the reference R moves faster than a point X in relation to the reference R, the interjacent material (red arrow) becomes stretched. In case of higher motion of Y in relation to R compared to Z in relation to R, the interjacent material becomes compressed (green arrows).

The following parameters of the spinal cord motion curve per heartbeat were generated per ROI: maximum velocity (cm/s), peak-to-peak (ptp)-amplitude (mm/s; maximum velocity–minimum velocity), total displacement (mm) (~area under the curve (AUC), but addition of inversed negative AUC values instead of subtraction) (Figure 2). Due to known moderate test–retest–reliability of the total displacement at segment C2, this single parameter was not considered [12].

In order to minimize effects of individual confounder on spinal cord motion (e.g., body size) and to analyze mechanical effects such as compression or stretching of interjacent spinal cord tissue, two indices were calculated: the C2-ptp-amplitude index (C2-pAI: [ptp-amplitude_(C3/4 − C7/T1)_ ÷ ptp-amplitude_C2/3_]) [12] and correspondingly, the C7-ptp-amplitude index (C7-pAI: [ptp-amplitude_(C2/3 − C6/C7)_ ÷ ptp-amplitude_C7/T1_]). The segments C2/C3 and C7/T1 are both suitable as references, as both have been reported to represent similar dynamics in healthy controls [11,12]. A cranio-caudal increase between two segments would indicate a mechanical stretch of the interjacent spinal cord tissue, whereas a cranio-caudal decrease would indicate a compression (Figure 2). As dynamics at adjacent segments to the stenosis were described to be altered as well [11,12], referencing was performed on the least affected, most remote segment. Thus, the C2-pAI (reference segment C2/C3) is suitable to gain information on strain mechanisms in case of caudal cervical stenosis, the C7-pAI (reference segment C7/T1), and vice versa. Indices provide a more sensitive inter-subject comparability.

### 2.5. Anatomical MRI-Parameters

The following anatomical parameters were automatically computed within the post-processing pipeline per segment: spinal cord CSA (mm^2^), spinal canal CSA (mm^2^), and the adapted maximum canal compromise (aMCC: [(spinal canal CSA one segment above + spinal canal CSA one segment below) ÷ (2 × spinal canal CSA at level)]) reflecting the severity of the individual’s spinal stenosis unrelated to body size [12,18]. In addition, we calculated an adapted spinal cord occupation ratio (aSCOR) in % per segment adapted to Nouri et al. [19] using the automatically generated CSAs of the spinal cord and the spinal canal per segment (spinal cord CSA × 100 ÷ spinal canal CSA). Thus, the aSCOR adds information on the segmental relationship of occupied spinal cord CSA to remaining cerebrospinal fluid (CSF)-space, which is not reflected by the aMCC.

### 2.6. Data Validity

Excellent data validity of the applied data processing and test-retest-reliability of all anatomical and dynamic data assessments (ICC > 0.9 [20]) has been reported before [12].

### 2.7. Statistics

Statistical analysis was conducted by SPSS Statistics® (IBM Corporation, Released 2020. IBM SPSS Statistics for Macintosh, Version 27.0. Armonk, New York, USA). Data is given as mean and standard deviation (SD). Quantitative data of patients and controls were compared segment by segment. Comparison of two groups, unrelated values, was conducted upon data distribution analysis (Shapiro-Wilk): normally distributed data was compared via t-test, non-normally distributed data via Mann–Whitney U-test. Comparison of multiple related variables was calculated via bonferroni-adjusted analysis of variance (ANOVA) with repeated measurements upon validation of distribution and sphericity; outliers were not excluded. Comparison of multiple unrelated variables was performed via Kruskal–Wallis Test. Prediction models were rated by multiple linear regressions upon validation of standard premises. Outliers were excluded if identified by two methods (Cook’s distance [21], leverage [22]). *p* was required to be <0.05 to assume significance.

## 3. Results

### 3.1. Study Population

Sixty-five patients were included in the final analysis (Table 1). Two presented with levels of stenosis at C2/C3 (50% men), six at C3/C4 (100% men), 14 at C4/C5 (64.3% men), 33 at C5/C6 (42.4% men), and 10 with levels of stenosis at C6/C7 (60% men). Therefore, due to the small number of participants, the majority of statistical analyses was not performed among patients with stenosis at C2/C3. A total of forty healthy age- and gender-matched controls were included in the study.

Age, mJOA score, number of patients receiving decompressive surgery, aSCOR, and aMCC per group are listed in Table 1. Patients with stenosis at C5/C6 (53 ± 12 years) and C6/C7 (54 ± 12 years) were significantly younger than patients with stenosis at C4/C5 (65 ± 9 years, *p* = 0.002, *p* = 0.021, respectively). Age did not differ between any other group of patients. Comparison of gender between groups showed no significant difference (*p* = 0.18–0.84), with exception of patients with stenosis at C3/C4 (100% men). Duration of the heartbeat was comparable between all groups of patients (*p* = 0.85) and between patients and controls (*p* = 0.25–0.78). The comparison of the mJOA score between groups of patients was not significant (C2/C3 vs. C6/C7, *p* = 0.078, any other comparison *p* > 0.5).

As expected, the aMCC at the stenotic level was significantly higher per group compared to controls (C2/C3 *p* = 0.026, C3/C4 *p* = 0.022, C4/C5, C5/C6, C6/C7 *p* < 0.001, respectively). The aMCC among patients with stenosis at C4/C5 (2.97 ± 0.2) was significantly higher compared to patients with stenosis at C3/C4 (1.96 ± 0.7, *p* = 0.002) and at C5/C6 (2.28 ± 0.9, *p* = 0.012). The aMCC between other groups did not differ.

aSCOR was expectedly significantly higher among patients (*p* ≤ 0.001, each), but within patients with stenosis at C2/C3 (*p* = 0.16).

### 3.2. Focal Increase of Spinal Cord Motion within All Groups of Patients

**Figure 3:**
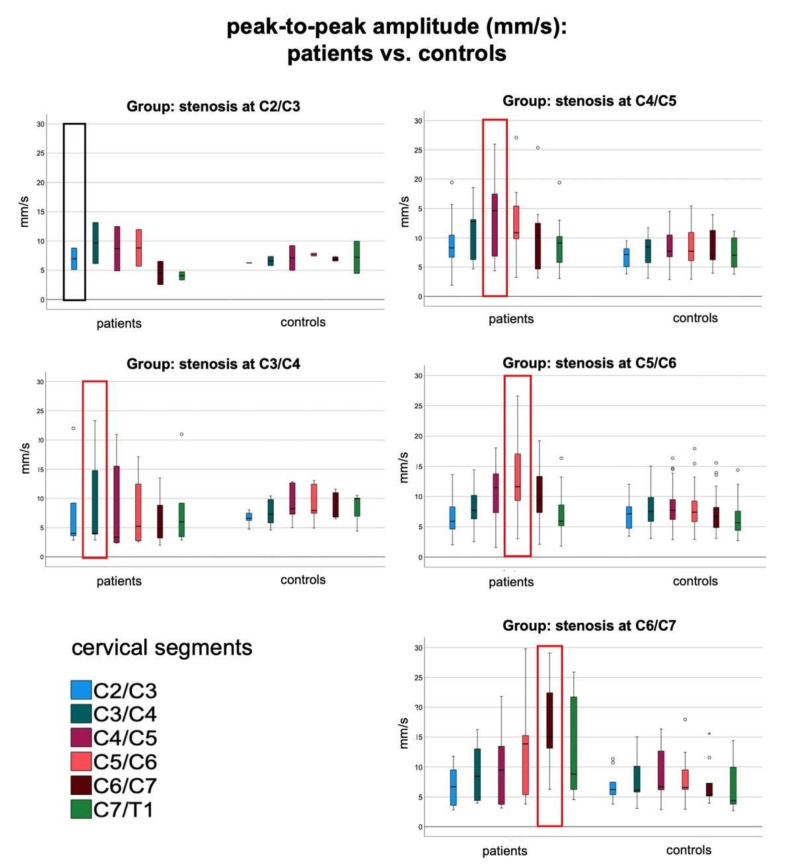
Boxplots per group of patients with level of stenosis at C2/C3, C3/C4, C4/C5, C5/C6, and C6/C7 compared to matched controls; the peak-to-peak amplitude of spinal cord motion is given in mm/s per cervical segment C2/C3 to C7/T1. Increase of spinal cord motion toward each level of stenosis (red rectangle) can be observed. Mild outliers are indicated by ° (1.5 to 3.0 × interquartile range), extreme outliers by * (>3.0 × interquartile range).

Compared to controls, patients with stenosis at C2/C3 and C3/C4 showed a trend toward higher spinal cord dynamics at stenosis and at the adjacent segments, but comparison did not reach significance (*p* > 0.5) (Figure 3, complete data sets in Table S1).

Spinal cord motion at level of stenosis was significantly higher among patients with stenosis at C4/C5, C5/C6, and C6/C7 (Figure 3, Table S1; e.g., ptp-amplitude (mm/s) − group C4/C5: 13.80 ± 6.7 mm/s vs. 7.94 ± 3.3 mm/s, *p* = 0.007; group C5/C6: 13.44 ± 6.4 mm/s vs. 7.89 ± 3.3 mm/s, *p* < 0.001; group C6/C7: 17.69 ± 7.5 mm/s vs. 7.31 ± 3.7 mm/s, *p* = 0.001). Similarly, the cranial and caudal adjacent segments showed significantly, or borderline significantly increased spinal cord dynamics (Figure 2, Table S1, e.g., ptp-amplitude (mm/s) one segment caudal to the stenosis: group C4/C5: 12.46 ± 6.1 vs. 8.07 ± 3.5, *p* = 0.02; group C5/C6: 9.95 ± 3.9 vs. 7.16 ± 3.1, *p* = 0.002; group C6/C7: 12.88 ± 7.9 vs. 6.57 ± 4.1, *p* = 0.038).

### 3.3. Mechanical Stretching and Compression of Interjacent Spinal Cord Tissue

**Figure 4:**
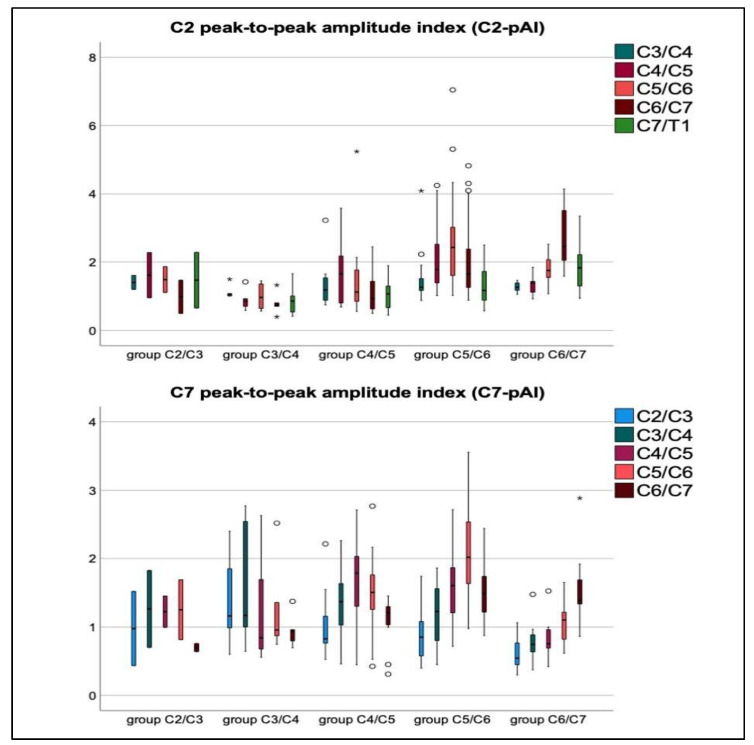
Boxplots of the C2-peak-to-peak amplitude index (C2-pAI, top row) and the C7-peak-to-peak amplitude index (C7-pAI, bottom row). The pAI references the spinal cord peak-to-peak amplitude per segment to the individual’s spinal cord peak-to-peak amplitude at the cranial segment C2/C3, or to the caudal segment C7/T1. Cranial levels of stenosis are best reflected by the C7-pAI, caudal stenosis by the C2-pAI. An increase (*y*-axis) toward the stenotic level per group (*x*-axis) followed by a decrease is shown, indicating a stretching of the spinal cord tissue cranial of the level of stenosis followed by a mechanical compression of tissue at the caudal segments. Increase and decrease were significant among the groups of patients with stenosis at C4/C5, C5/C6, and C6/C7. Mild outliers are indicated by ° (1.5 to 3.0 × interquartile range), extreme outliers by * (>3.0 × interquartile range).

Compared to controls, indices (pAI) at stenosis were significantly increased among patients with stenosis at C4/C5, C5/C6, and C6/C7 (Figure 4).

Patients with levels of stenosis at C4/C5 showed a significant decrease of the C2-pAI from C4/C5 to C6/C7 (*p* = 0.011), highlighting a primarily caudal compression of the spinal cord tissue (Table S1, Figure 4). Patients with stenosis at C5/C6 and C6/C7 showed a significant increase from C2/C3 toward stenosis, and a significant decrease from stenosis to C7/T1 (increase: *p* ≤ 0.001, each, decline: *p* = 0.019–0.036, respectively; Table S1, Figure 4). Among patients with levels of stenosis at C2/C3 and C3/C4 comparison of indices showed a non-significant trend toward higher values at stenosis (Table S1).

### 3.4. Relations of Severity of Stenosis (aMCC/aSCOR), Age, Gender, and mJOA Score to Increased Spinal Cord Motion at Stenosis

Prediction models of each spinal cord motion parameter at stenosis by (1: aMCC at stenosis, age, gender) or (2: aSCOR at stenosis, age, gender) were calculated within all suitable groups of patients with stenosis at C3/C4, C4/C5, C5/C6, and C6/C7. Gender as a predictor could not be analyzed among patients with stenosis at C3/C4 (100% men).

aMCC, aSCOR, age, and mJOA score did not reach significance within any prediction models. One model (1: aMCC at stenosis, age, gender) significantly predicted the C2-pAI at stenosis among patients with stenosis at C6/C7 (1:R^2^ = 0.933, *p* = 0.042), gender being the only significant predictor (*p* = 0.033).

Gender was a significant predictor of higher spinal cord motion at stenosis among men in group C5/C6 (1: *p* = 0.048; higher ptp-amplitude), and of higher spinal cord strain among men in group C6/C7 (1: *p* = 0.033, 2: *p* = 0.024; higher C2-pAI).

**Figure 5:**
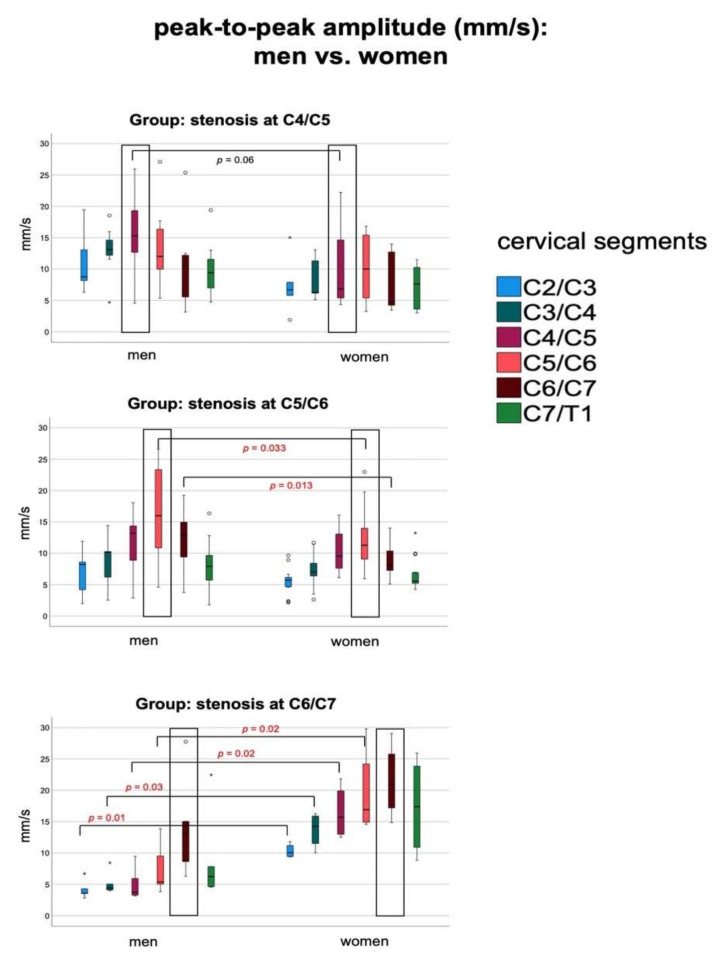
Boxplots demonstrating differences of peak-to-peak amplitudes (mm/s) between men and women. Level of significance is provided in brackets, borderline significance in black lettering, and significant difference in red lettering. The typical increase toward levels of stenosis followed by a decrease can be observed among all groups (black rectangle). Men with stenosis at C6/C7 show a significant decrease of spinal cord motion prior to the stenotic segment. Mild outliers are indicated by ° (1.5 to 3.0 × interquartile range), extreme outliers by * (>3.0 × interquartile range).

The comparison of spinal cord motion between men and women per group revealed increased maximum velocity and/or ptp-amplitudes at stenosis among patients with stenosis at C4/C5 and C5/C6 (*p* = 0.03, *p* = 0.064, and *p* = 0.03, *p* = 0.028, respectively; Figure 5). Total displacement did not differ between men and women in these groups (*p* = 0.89, *p* = 0.25, respectively; Table 2).

In contrast, men with stenosis at C6/C7 revealed atypically decreased spinal cord motion at segments cranial to the stenosis. Comparison to matched controls (all parameter, *p* = 0.009 to 0.027, Figure 5, Table 2), and to women with stenosis at C6/C7 (*p* < 0.001 to 0.02) showed significantly lower values. At the level of stenosis, spinal cord motion did not differ between genders. There was no significant difference of age, HB, mJOA score, spinal canal CSA, aSCOR, or aMCC between men and women per group with stenosis at C4/C5, C5/C6, or C6/C7, nor between men or women with stenosis at C5/C6 and men with stenosis at C6/C7.

## 4. Discussion

To date, our work represents the most extensive study on spinal cord motion demonstrating focal and long distant pathodynamic patterns in DCM patients while covering relevant stenoses at all cervical segments. Moreover, this is the first report on significant dynamic alterations due to gender-related effects among DCM patients. Additionally, our data suggest segmental differences of spinal cord motion behavior depending on the stenotic cervical segment and therefore underlines the importance of focal influences on spinal cord motion.

We report on 65 DCM patients presenting with monosegmental stenosis. Our study population represents a common, clinically mildly affected cohort, with C5/C6 being the most commonly stenotic segment [23]. Thus, the cohort consists of a representative sample that typically would require additional diagnostics during medical workup.

The current data replicates the dynamic alterations already observed at C5/C6 also at other stenotic cervical segments [10,12,13]. This is of interest, as the segment C5/C6 is located at the maximum of the cervical lordosis and healthy controls show a physiological increase of spinal cord motion at this segment [11,12]; Figure 3. Current results show significant differences between DCM patients and matched-paired controls specifically depending on the level of the stenotic segment: focally increased spinal cord motion appears with a maximum at stenosis and remains relatively unaffected at segments remote from stenosis. The intra-individual indices replicate an overall strain on spinal cord tissue among patients with stenoses at other cervical levels [12]. Among all larger groups of patients, a significant decrease of the indices below level of stenosis was demonstrated as well, indicating an additional compressive effect on the spinal cord tissue caudal of the stenosis. This relative decrease of spinal cord motion below the stenotic segment may be an effect of higher pressure within the spinal canal caudal of the stenosis, following the law of Bernoulli.

Consistent with earlier findings [10,12], automated measurements of the severity of the stenosis (aMCC, aSCOR) did not predict the extent of spinal cord motion, emphasizing a focal disarrangement of spinal cord dynamics and anatomy. The generally suspected origin of spinal cord motion has been extensively discussed previously [10,11,12,13]. In summary, known influences on intraspinal dynamics can be divided in global (e.g., heartbeat [24,25,26]), breathing [27], and pulsatile CSF-flow [28]) and focal effects, such as loss of compensatory buffer zone for the expansion of pulsatile local arteries [28]).

The demonstrated missing relationship of spinal cord motion to the severity of stenosis may possibly be due to differences of the capacity to compensate for alterations of pulsatile subarachnoid CSF-pressure changes within the spinal canal-analogous to the Windkessel effect. Thus, as there exists a well-known variance between clinical impairment and severity of spinal canal compression [1,5,6,7], spinal cord motion may be a possible predictor of the clinical course in case of spinal canal stenoses.

Although current data show similar spinal cord motion patterns across all groups of patients with different levels of stenosis, current data may imply differences between men and women.

While the velocity peaks of the spinal cord motion curve over one heartbeat were significantly increased among men compared to women with stenosis at C4/C5 and C5/C6 (maximum velocity, peak-to-peak amplitude), the total displacement—a parameter comprising information of the entire velocity curve—remained similar between genders. As the mean duration of the heartbeat was not different between men and women, a lower peak but with similar total displacement indicated a flattened peak and a prolonged sinusoidal spinal cord motion curve over one heartbeat among women. This finding is complementary to the recently described spinal cord motion curve pattern among DCM patients by Hupp et al. In contrast to controls with a short, singular spinal cord oscillation within the heart cycle, DCM patients showed an ongoing spinal cord motion during the entire heart cycle [13]. Among women, this effect seems to be intensified.

As an unexpected, possibly gender-related effect, a uniquely altered spinal cord motion pattern was observed among men with stenosis at C6/C7: Spinal cord motion was significantly slower at segments cranial to the stenosis followed by a vast acceleration at stenosis. Sufficient explanation for this observation cannot be concluded based on the currently assessed data (age, spinal canal CSA, aSCOR, etc.), that did not differ between genders or groups. As the assessed parameters on spinal canal anatomy and age did not significantly differ between genders, possible compensatory mechanisms as elasticity of meninges leading toward differences of volume–compensation within the subarachnoid CSF-space may play a role. The possibility of gender effects within DCM is underlined by significantly worse functional outcome of men undergoing decompressive surgery, which has been recently reported [29].

The only other trial on spinal cord motion across all cervical segments in 55 DCM patients with mono- (*n* = 19) and multisegmental (*n* = 36) stenoses based on validated analysis procedures was recently published by Hupp et al. [13]. Due to combined analysis of spinal cord motion pattern at different segments and non-matched cohorts, a point-to-point comparison is difficult. As a topic of inter-scanner and inter-protocol comparability, the currently reported velocity values among patients and controls based on different PC-MRI settings are at a higher level (approximately × 2) [12,13]. Further investigations should evaluate the inter-scanner reliability. As common ground, both studies underline pathological alterations of spinal cord motion pattern among DCM patients and its possible contributing value in DCM diagnostics. The currently presented data offers more refined results and depicts relevant differences of the pathodynamics per cervical segment between men and women. Thus, further studies should aim to investigate multicentric data and the clinical value of spinal cord motion based on segmental analysis.

Limits of the study are in part small cohorts (group C2/C3, C3/C4) and sub-cohorts. This is mostly due to the known contribution of most to least affected segments in DCM, but also to the exclusion of multisegmental relevant stenoses. At the current state of basic research and the general aim of an understanding of intrathecal dynamics, we chose to limit the possible confounding effect of multisegmental stenosis.

Effects on (yet) non-symptomatic spinal stenosis were not a topic of this study and should be investigated in further longitudinal trials. Differences in spinal canal degeneration, e.g., rather soft disc herniation vs. solid ossification of ligaments, and their possible influence on spinal cord dynamics were not systematically addressed. The study does not include a full analysis of physiological relations (e.g., body mass indices and height), nor associations to clinical function due to primary aims on pathophysiological ground research. Due to small group size, we cannot sufficiently analyze the association of clinical impairment to spinal cord motion based on the current data. The mJOA score reflects the range of patients included within the presented data, but it does not comprise details on mild spinal cord affection. First, more refined and reliable clinical and electrophysiological assessments are needed to assess the many aspects of DCM (e.g., reliable light-touch, pin-prick testing as part of the International Standards for Neurological Classification of Spinal Cord Injury [30] evoked potentials [31], the graded redefined assessment of strength, sensibility, and prehension version myelopathy [32], etc.). Second, more knowledge of the influencing factors is required in respect to gender and level of the cervical segment in order to establish reliable and comparable cut-off values.

## 5. Conclusions

The presented data presents focally increased spinal cord motion depending on the affected cervical stenotic segment among DCM patients. There is proof of an overall disarranged dynamic behavior resulting in mechanical strains on spinal cord tissue across the cervical spine. Men and women show significantly different spinal cord motion patterns depending on the affected cervical segment, thus indicating gender-related differences in DCM.

## Figures and Tables

**Table 1:** Study-population.

	Level of Stenosis	C2/C3	C3/C4	C4/C5	C5/C6	C6/C7
Patients	*n*	2	6	14	33	10
	Male (%)	1 (50)	6 (100)	9 (64.3)	14 (42.4)	6 (60)
	age (years) (mean ± SD)	57 ± 8	64 ± 10	65± 9	53 ± 12	54 ± 12
	mJOA (mean ± SD)	18	14.50 ± 3.2	15.85 ± 2.2	16.47 ± 1.8 *	15.4 ± 2.1
	mJOA 18 (%)	1 (50)	1 (16.7)	5 (35.7)	10 (31.3)	1 (10)
	mJOA 15–17 (%)	1 (50)	3 (50)	7 (50)	14 (43.8)	5 (50)
	mJOA < 15 (%)		2 (33.3)	2 (14.4)	7 (21.9)	4 (40)
	Surgical treatment (%)	0	3 (50)	8 (57.1)	18 (54.5)	6 (60)
	aMCC (mean ± SD)	2.24 ± 0.3	1.96 ± 0.7	2.97 ± 1.2 **	2.28 ± 0.9	2.20 ± 0.6
	aSCOR % (mean ± SD)	60 ± 20	74 ± 18	84 ± 9	83 ± 14	79 ± 10
Controls age- & gender- matched pairs	*n*	2	6	14	33	10
	Male (%)	1 (50)	6 (100)	9 (64.3)	13 (39.4)	6 (60)
	age (years, mean ± SD)	58 ± 8	64 ± 8	66 ± 9	54 ± 12	55 ± 12
	*p*	0.909	0.937	0.874	0.934	0.796
	aMCC (mean ± SD)	0.95 ± 0.1	1.12 ± 0.1	1.13 ± 0.1	1.21 ± 0.1	1.15 ± 0.1
	*p*	0.026	0.022	<0.001	<0.001	<0.001
	aSCOR % (mean ± SD)	30 ± 2	36 ± 5	37 ± 5	41 ± 7	37 ± 8
	*p*	0.158	0.001	<0.001	<0.001	<0.001

mJOA, modified Japanese Orthopedic Association (score); aMCC, adapted maximum cord compression; aSCOR, adapted spinal cord occupation ratio; SD, standard deviation; * incomplete data mJOA in two patients, total of *n* = 31; ** significantly higher compared to patients with stenosis at C3/C4 (*p* = 0.002) and compared to patients with stenosis at C5/C6 (*p* = 0.012).

**Table 2:** Clinical, anatomical, and spinal cord motion data per suitable group of patients divided by gender.

		C4/C5	C5/C6	C6/C7
Age (years) (mean ± SD)	men	65 ± 8	53 ± 13	51 ±9
	women	66 ± 11	54 ± 11	59 ± 16
	*p*	0.819	0.875	0.345
mJOA (mean ± SD)	men	15.9 ± 1.9	16.5 ± 1.7	15.5 ± 2.1
	women	17.0 ± 0.8	16.3 ± 1.9	15.3 ± 2.4
	*p*	0.289	0.756	0.864
HB (ms) (mean ± SD)	men	944 ± 109	926 ± 138	857 ± 172
	women	962 ± 168	910 ± 134	934 ± 229
	*p*	0.827	0.732	0.761
aMCC at stenosis (mean ± SD)	men	2.8 ± 1.1	2.6 ± 1.2	2.5 ± 0.6
	women	3.3 ± 1.3	2.0 ± 0.7	1.8 ± 0.3
	*p*	0.468	*0.084*	*0.065*
aSCOR % at stenosis (mean ± SD)	men	83.0 ± 9	82.9 ± 11	82.6 ± 12
	women	85.0 ± 10	82.7 ± 16	73.3 ± 5
	*p*	0.72	0.968	0.182
Max. velocity (cm/s) at stenosis (mean ± SD)	men	1.00 ± 0.5	1.09 ± 0.6	0.94 ± 0.6
	women	0.59 ± 0.4	0.73 ± 0.4	1.49 ± 0.4
	*p*	**0.03**	**0.03**	0.13
ptp-amplitude (mm/s) at stenosis (mean ± SD)	men	15.5 ± 5.9	16.3 ± 7.1	15.2 ± 7.9
	women	10.7 ± 7.6	11.4 ± 5.1	21.5 ± 5.9
	*p*	*0.064*	**0.028**	0.211
Total displacement (mm) at stenosis (mean ± SD)	men	1.92 ± 0.9	1.92 ± 0.9	1.7 ± 0.6
	women	1.83 ± 1.5	1.49 ± 0.9	2.4 ± 0.9
	*p*	0.893	0.255	0.151
C2-pAI at stenosis (mean ± SD)	men	1.56 ± 0.7	2.76 ± 1.1	3.23 ± 0.9
	women	1.79 ± 1.3	2.45 ± 1.5	2.05 ± 0.4
	*p*	0.682	0.523	**0.046**
C7-pAI at stenosis (mean ± SD)	men	1.79 ± 0.8	2.21 ± 0.7	1.71 ± 0.6
	women	1.54 ± 0.6	1.96 ± 0.6	1.35 ± 0.4
	*p*	0.570	0.31	0.33

mJOA, modified Japanese Orthopedic Association (score); HB, heartbeat; aMCC, adapted maximum cord compression; aSCOR, adapted spinal cord occupation ratio; Max, maximum; ptp, peak-to-peak; pAI, peak-to-peak amplitude index. Significant differences in bold letters, borderline significance in italic letters.

## Data Availability

The data presented in this study are openly available in Table S1, and Table 1 and Table 2.

## Supplementary Materials

The following are available online at https://www.mdpi.com/article/10.3390/jcm10173788/s1, Table S1: Table of all means, standard deviations, and *p* values of all dynamic parameters per segment and group.

## Abbreviations

aMCC	adapted maximum canal compromise
aSCOR	adapted spinal cord occupation ratio
CNN	convolutional neural network
CSA	cross-sectional area
CSF	cerebrospinal fluid
DCM	degenerative cervical myelopathy
ICC	intra-class correlation coefficient
MRI	magnetic resonance imaging
pAI	peak-to-peak-amplitude index
PC-MRI	phase-contrast MRI
ptp	peak-to-peak
SPACE	sampling perfection with application-optimized contrasts using different flip angle evolution
