# Supplementary material for: Spinal Cord Motion in Degenerative Cervical Myelopathy: The Level of the Stenotic Segment and Gender Cause Altered Pathodynamics
Source: J Clin Med. 2021 Aug 25;10(17):3788. doi: 10.3390/jcm10173788 (PMC8432264; doi:10.3390/jcm10173788)
Supplement: Supplementary file 1 [file jcm-10-03788-s001.zip › jcm-1322054-supplementary.pdf]

Table S1. Spinal cord motion data per group and segment.

Group	Segment		Max. Velocity (cm/s)			ptp-Amplitude (mm/s)			Total Displacement (mm)			C2-pAI			C7-pAI		
			Mean	SD	*p*	Mean	SD	*p*	Mean	SD	*p*	Mean	SD	*p*	Mean	SD	*p*
C2/C3	C2/C3	patients	0.48	0.2	0.728	6.95	2.6	0.741							1.69	0.2	0.255
		controls	0.42	0.1		6.26	0.0								1.02	0.6	
	C3/C4	patients	0.71	0.3	0.372	9.66	5.0	0.482	0.54	0.3	0.071	1.35	0.2	0.255	2.30	0.7	0.237
		controls	0.43	0.1		6.58	1.1		1.21	0.0		1.05	0.2		1.11	0.7	
	C4/C5	patients	0.54	0.2	0.766	8.70	5.4	0.750	0.67	0.3	0.246	1.19	0.3	0.906	2.04	0.8	0.518
		controls	0.47	0.2		7.11	3.0		0.98	0.1		1.13	0.5		1.28	1.1	
	C5/C6	patients	0.51	0.1	0.831	8.83	4.4	0.752	1.13	0.7	0.697	1.24	0.2	0.959	2.11	0.6	0.329
		controls	0.49	0.0		7.69	0.3		1.35	0.0		1.23	0.0		1.26	0.7	
	C6/C7	patients	0.29	0.2	0.439	4.55	2.8	0.358	0.52	0.1	0.175	0.62	0.2	0.070	1.07	0.4	0.949
		controls	0.43	0.0		6.94	0.5		1.22	0.5		1.11	0.1		1.10	0.5	
	C7/T1	patients	0.23	0.1	0.480	4.06	1.0	0.381	0.56	0.1	0.228	0.60	0.1	0.340			
		controls	0.42	0.3		7.21	3.9		1.53	0.8		1.15	0.6				
C3/C4	C2/C3	patients	0.61	0.6	0.472	8.33	8.0	0.651							1.12	0.8	0.525
		controls	0.42	0.1		6.62	1.3								0.86	0.4	
	C3/C4	patients	0.68	0.6	0.342	9.53	8.1	0.480	1.15	0.8	0.869	1.15	0.3	1.000	1.25	0.7	0.493
		controls	0.43	0.1		6.98	2.7		1.10	0.2		1.12	0.2		1.02	0.4	
	C4/C5	patients	0.55	0.5	0.945	9.17	7.8	0.831	1.12	0.8	0.927	1.07	0.7	0.310	1.10	0.5	0.701
		controls	0.53	0.2		8.39	3.7		1.09	0.2		1.38	0.4		1.21	0.4	
	C5/C6	patients	0.44	0.3	0.734	8.14	5.8	0.886	1.19	0.7	0.902	1.10	0.6	0.400	1.04	0.3	0.408
		controls	0.50	0.2		8.55	3.5		1.15	0.4		1.35	0.3		1.24	0.5	
	C6/C7	patients	0.39	0.2	0.853	6.81	4.3	0.627	0.92	0.4	0.342	0.94	0.4	0.147	0.92	0.3	0.271
		controls	0.42	0.2		7.87	2.9		1.14	0.3		1.30	0.2		1.11	0.3	
	C7/T1	patients	0.45	0.4	0.847	8.04	6.7	0.866	1.34	1.2	0.813	1.25	0.7	0.938			
		controls	0.42	0.2		7.51	3.2		1.21	0.5		1.28	0.4				
C4/C5	C2/C3	patients	0.49	0.2	0.761	9.46	4.7	0.071							1.03	0.5	1.000
		controls	0.46	0.2		6.73	1.7								0.99	0.4	

	C3/C4	patients	0.70	0.2	0.017	11.16	4.3	0.011	1.47	0.7	0.040	1.34	0.6	0.574	1.34	0.6	0.376
		controls	0.48	0.2		7.40	2.6		1.07	0.2		1.13	0.2		1.16	0.4	
	C4/C5	patients	0.85	0.5	0.030	13.80	6.7	0.007	1.88	1.1	0.012	1.65	0.9	0.160	1.68	0.7	**0.032**
		controls	0.52	0.2		7.94	3.3		1.04	0.3		1.23	0.3		1.21	0.3	
	C5/C6	patients	0.77	0.4	0.033	12.46	6.1	0.028	1.71	1.0	0.054	1.56	1.2	0.769	1.54	0.6	0.180
		controls	0.48	0.3		8.07	3.5		1.06	0.3		1.22	0.3		1.24	0.4	
	C6/C7	patients	0.58	0.4	0.274	9.61	5.9	0.701	1.32	0.8	0.943	1.09	0.6	0.496	1.11	0.4	1.000
		controls	0.45	0.2		7.89	3.1		1.16	0.4		1.22	0.3		1.20	0.3	
	C7/T1	patients	0.51	0.3	0.167	8.82	4.2	0.157	1.38	0.8	0.309	1.05	0.4	0.673			
		controls	0.39	0.2		6.85	2.8		1.12	0.5		1.11	0.3				
	C2/C3	patients	0.42	0.2	0.341	6.30	2.9	0.390							0.96	0.6	**0.009**
		controls	0.47	0.2		6.86	2.3								1.20	0.5	
	C3/C4	patients	0.54	0.2	0.890	8.07	3.1	0.879	1.07	0.4	0.837	1.40	0.6	0.047	1.26	0.6	0.263
		controls	0.53	0.2		7.96	2.9		1.06	0.2		1.16	0.2		1.40	0.6	
	C4/C5	patients	0.66	0.3	0.039	10.47	4.0	0.011	1.38	0.6	0.003	1.95	0.9	0.000	1.60	0.6	0.148
C5/C6		controls	0.52	0.2		8.09	3.3		1.02	0.3		1.18	0.3		1.40	0.5	
	C5/C6	patients	0.88	0.5	0.000	13.44	6.4	0.000	1.68	1.0	0.001	2.51	1.4	0.000	2.04	0.7	**0.000**
		controls	0.50	0.2		7.89	3.3		0.99	0.3		1.16	0.3		1.34	0.5	
	C6/C7	patients	0.62	0.3	0.002	9.95	3.9	0.002	1.31	0.6	0.026	1.95	1.1	0.000	1.49	0.4	**0.004**
		controls	0.42	0.2		7.16	3.1		1.03	0.3		1.07	0.4		1.20	0.3	
	C7/T1	patients	0.41	0.2	0.287	7.06	3.2	0.295	1.08	0.4	0.595	1.29	0.5	0.008			
		controls	0.36	0.2		6.27	2.7		1.03	0.4		0.95	0.3				
	C2/C3	patients	0.47	0.3	0.867	6.91	3.5	1.000							0.62	0.2	**0.019**
		controls	0.49	0.2		6.91	2.7								1.29	0.7	
	C3/C4	patients	0.58	0.3	0.559	9.04	4.7	0.555	1.22	0.6	0.652	1.28	0.1	0.174	0.78	0.3	**0.015**
		controls	0.50	0.3		7.92	3.5		1.12	0.3		1.15	0.2		1.46	0.7	
C6/C7	C4/C5	patients	0.68	0.5	0.420	10.43	6.5	0.394	1.26	0.7	0.333	1.35	0.3	0.295	0.84	0.3	**0.030**
		controls	0.54	0.3		8.29	4.3		1.03	0.3		1.20	0.3		1.55	0.9	
	C5/C6	patients	0.90	0.6	0.054	13.32	7.9	0.082	1.56	0.8	0.060	1.79	0.4	0.003	1.08	0.3	0.141
		controls	0.50	0.3		8.09	4.3		1.02	0.3		1.17	0.3		1.43	0.6	
	C6/C7	patients	1.16	0.6	0.001	17.69	7.5	0.001	1.99	0.8	0.002	2.71	0.9	0.000	1.57	0.5	0.216

	controls	0.43	0.2		7.31	3.7		1.01	0.3		1.05	0.3		1.28	0.5
C7/T1	patients	0.79	0.5	0.037	12.88	7.9	0.038	1.59	0.8	0.071	1.84	0.7	0.008		
	controls	0.39	0.3		6.57	4.1		1.00	0.6		0.98	0.4			

Max.: maximum, ptp: peak-to-peak, pAI: peak-to-peak amplitude index = peak-to-peak amplitude per segment referenced to the individual's peak-to-peak amplitude at C2/C3 (C2-pAI) or C7/T1 (C7-pAI) ; SD: standard deviation. Significant differences ($p < 0.05$) are indicated in bold lettering.
